# *HLA’s* hidden hand in Alzheimer’s disease—five research questions en route to an answer

**DOI:** 10.1093/braincomms/fcaf438

**Published:** 2025-11-08

**Authors:** Lorenzo Capitani, Sarah M Carpanini

**Affiliations:** UK Dementia Research Institute, School of Medicine, Cardiff University, Hadyn Ellis Building, Maindy Road, Cardiff CF24 4HQ, UK; Division of Infection and Immunity, School of Medicine, Cardiff University, Henry Wellcome Building, Heath Park, Cardiff, CF14 4XN, UK; UK Dementia Research Institute, School of Medicine, Cardiff University, Hadyn Ellis Building, Maindy Road, Cardiff CF24 4HQ, UK; Division of Infection and Immunity, School of Medicine, Cardiff University, Henry Wellcome Building, Heath Park, Cardiff, CF14 4XN, UK

**Keywords:** Alzheimer’s disease, human leukocyte antigen, T cells, genetics

## Abstract

The association of genetic variants in the Human Leukocyte Antigen (*HLA*) locus with late-onset Alzheimer’s disease has been stringently replicated across several, powerful genome-wide association studies. However, no clear picture has yet emerged of the mechanistic relationship between Alzheimer’s disease and this top genetic hit, despite the fact that the *HLA* locus is one of the most influential gene loci of the immune system, known to influence antigen presentation, T cell responses and brain plasticity. In this review, we explore this association by outlining five research questions, namely: (i) the association of *HLA* Class I and Class II genes with Alzheimer’s disease at the allelic and haplotypic levels, (ii) the unconventional role of *HLA* Class I in the brain, (iii) the infection hypothesis of Alzheimer’s disease in the context of the known role HLA proteins play in immunity, (iv) the possible antigen presentation of Alzheimer’s disease relevant self-antigens and in turn (v) the possibility of T cells existing that are specific for these antigens. Identifying the functional mechanisms underlying this important genetic association with Alzheimer’s disease may hold the key to unravelling new avenues of Alzheimer’s disease immunotherapeutics.

## Teaser

Capitani et al. review the relationship between the Human Leukocyte Antigen system and Alzheimer’s disease, presenting five perspectives; genetic association, *HLA* class I in the brain, infection hypothesis, antigen presentation and T-cell responses. Understanding the link between Human Leukocyte Antigen and Alzheimer’s disease may open the door to new immunotherapeutics.

## Graphical Abstract

**Graphical Abstract:**
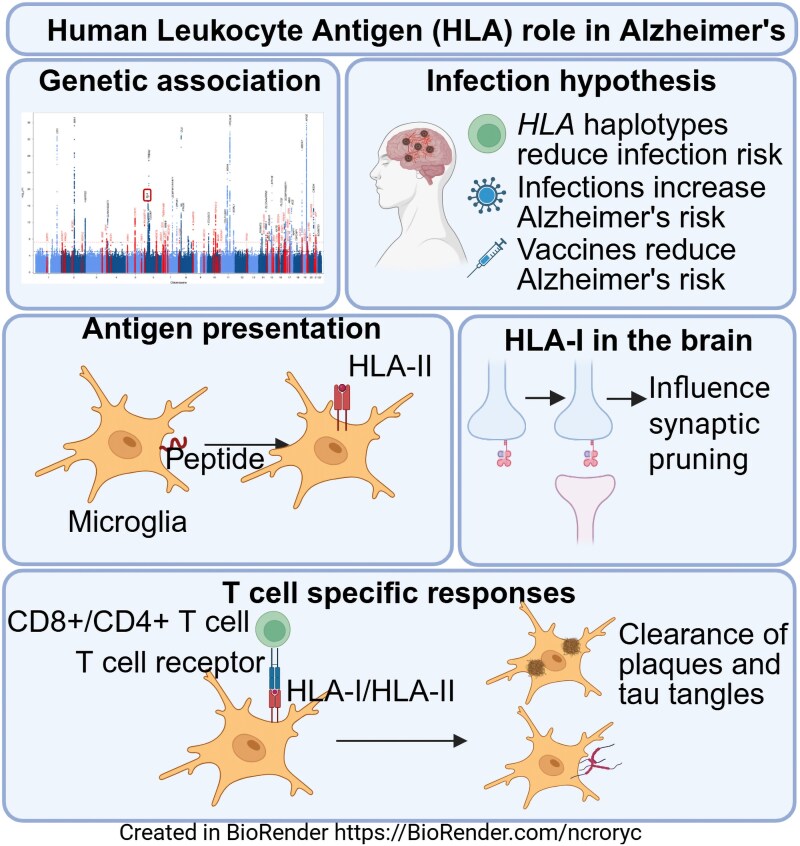

## Introduction

The brain is a complex organ, long thought to be a site devoid of immune activity. This gave the organ it's infamous title of ‘immune privileged’, a badge earned in part, due to the (falsely) believed absence of lymphatic drainage, tight blood-brain barrier and the limited rejection of peripheral tissues transplanted into the brain.^1^ However, in recent years, our understanding of the involvement of the immune system in brain physiology has soared, and it is now recognized that components of the immune system play an active role within the brain. For example, myeloid-derived microglia are resident in the brain and, amongst their numerous roles is pruning synapses during development, driven, at least in part, by the complement system.^2^ Beyond this, microglia perform a protective role in maintaining local homeostasis, capable of producing a variety of factors like TNF-α, IL-6 and IFN-γ to modulate the maturation and survival of surrounding oligodendrocytes^3-5^ whilst also being involved in protection against infection, particularly against neurotropic viruses.^6^

As medicine advances, and the average human life-span increases,^7^ neurodegenerative diseases like dementia are becoming an increasingly common societal burden. Alzheimer’s disease (AD) is the most common form of dementia, characterized by the accumulation of amyloid-β (Aβ), a normal cellular product resulting from the metabolism of amyloid precursor protein (APP) and neurofibrillary tangles composed of hyperphosphorylated tau. The first hints of immune involvement in AD stemmed from the identification of alterations in glial cells in Alois Alzheimer’s first report,^8^ the subsequent discovery of immunoglobulin fragments binding to amyloid plaques^9^ and the exacerbation of cognitive decline in AD after systemic infection.^10^ Since then, it has become widely established that neuroinflammation and microglial activation play a driving role in neuronal loss and AD pathology rather than merely a consequence of disease.^11^ Genetic studies have shown that numerous genes involved in inflammation and immunity, including human leukocyte antigen (*HLA*) genes, associate with AD risk.^12^ Herein, we outline five evolving research questions directed at understanding how one of the most pivotal genetic loci of the adaptive immune system alters disease risk.

### *HLA* allele associations—which of the many?

**Figure 1:**
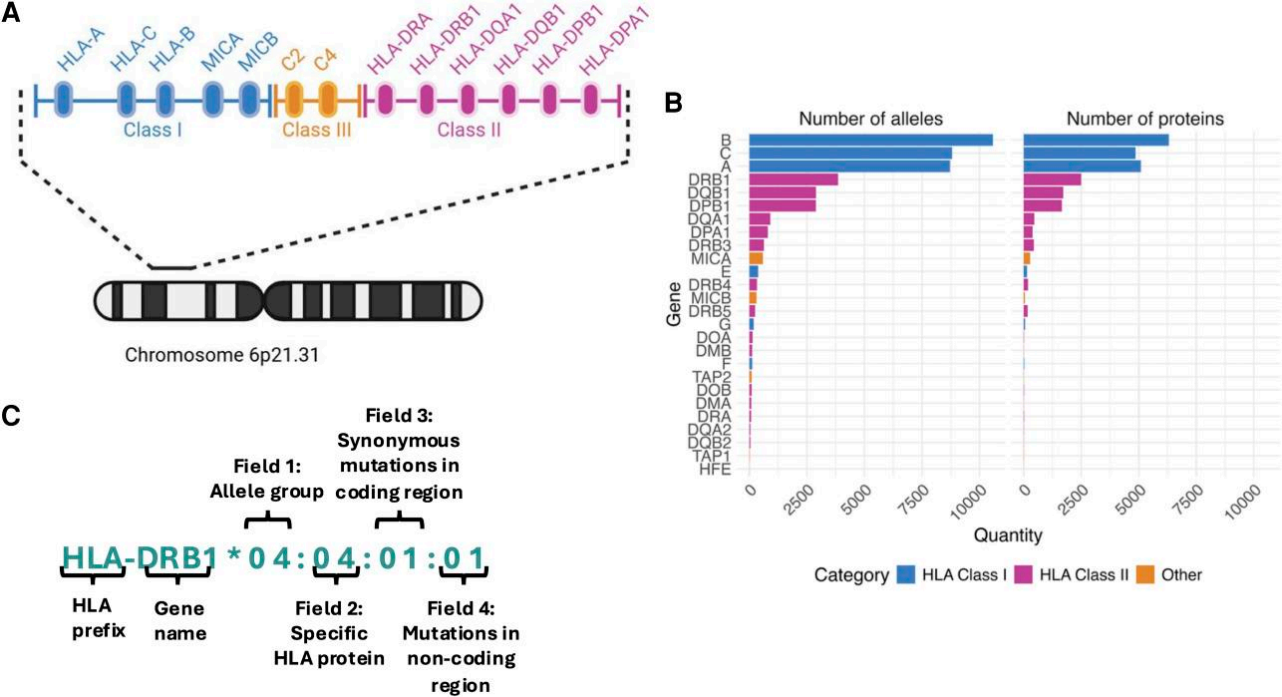
**Unpacking the complexity of the HLA gene locus.** (**A**) Summary schematic of the organization of the HLA gene locus on chromosome 6. The HLA Class I locus is represented in blue. The HLA Class II locus is represented in pink. Finally, the HLA Class III region, which does not encode classical HLA proteins, instead encoding inflammatory molecules like C2, is represented in orange. For simplicity not all genes in each locus are represented. (**B**) Number of alleles identified to date for each HLA Class I and Class II gene based on data available on the ImMunoGeneTics (IMGT) HLA database release version 3.6.1, highlighting the extreme degree of polymorphism of each gene in the HLA gene locus.^13,14^ (**C**) Breakdown of the four-field nomenclature for HLA alleles as defined by the ‘WHO Nomenclature Committee for Factors of the HLA System’. Typically, association studies like those in Table 1 and Table 2 employ two field resolution (i.e. reporting Fields 1 and 2), also known as 4-digit resolution. Figure 1A Created in BioRender. Carpanini, S. (2025) https://BioRender.com/w7h5tzw.

The major histocompatibility complex (MHC) region found on human chromosome 6p21 is the most polymorphic region of the human genome which is subdivided into three regions: the classical class I, class II and class III regions. The classical class I subregion contains canonical HLA Class I (*HLA-I*) genes like *HLA-A, HLA-B* and *HLA-C*. Similarly, the classical class II subregion contains canonical HLA Class II (*HLA-II*) genes like *HLA-DRB1, HLA-DQB1* and *HLA-DPB1*. Finally, the classical class III subregion contains non-HLA genes like *C2* and *C4* (Fig. 1A). *HLA-I* and *II* genes have arisen via gene duplication and divergence from a common ancestor,^15^ and it is now recognized that there are thousands of different alleles within this locus, many differing at the protein level by only one amino acid (Fig. 1B). Given this, a standardized method of nomenclature for naming *HLA* alleles has been established (Fig. 1C). *HLA* genes are tightly linked and inherited together as *HLA* haplotypes due to the strong linkage disequilibrium in the region and some *HLA* haplotypes are found more frequently in certain populations. The complexity and diversity of the *HLA* genes are essential for the immune system’s ability to present peptides to T cells from an immense variety of pathogens, including pathogens which have not yet evolved. This high degree of polymorphism is maintained by the necessity to display an array of peptides to T-cell receptors during the immune response. As such, the frequency of different alleles varies in diverse populations due to pathogen-driven selection.^16^

Although *HLA* association with AD risk was hypothesized at the end of the 1970s,^17^ genome-wide association studies (GWAS) have now reported compelling evidence of AD risk variants present within the *HLA* gene locus, implicating HLA biology, and in turn T cell responses, in the development of late-onset AD (>65 years).^12^ HLA focused studies employing methods of *HLA* typing, including PCR amplification-based methods or *HLA* imputation from single nucleotide polymorphism (SNP) microarrays, have also reported similar associations at the allelic level.^18-20^ Certain *HLA* alleles and haplotypes have reported to associate with protection against AD development.^12,18,21,22^ In Table 1 (Protective) and Table 2 (Risk), we have collated studies that have tested the association of *HLA* with AD at the level of individual *HLA* alleles (see Fig. 1C for a breakdown of *HLA* nomenclature), as opposed to SNP variants, given these are more easily interpretable and comparable to one another.

**Table 1:** Protective HLA haplotypes associated with AD

HLA allele	Population	Cohort size	OR^a^	Reference
HLA-A*01	Italian	173 cases & 258 controls		Guerini *et al*.^20^
HLA-DRB1*13:02	Western European	Correlation of population statistics		James and Georgopoulos^23^
HLA-DRB1*04 (04:04, 04:07, 04:03, 04:01)	Ethically diverse	121 411 cases & proxy and 409 096 controls	0.86, 0.88, 1.09, 0.93	Le Guen *et al*.^18^
HLA-DRB1*04	Iranian	145 cases & 101 controls		Rezaei *et al*.^24^
HLA-DRB1*09	Caucasian (U.K.)	48 cases & 44 controls		Neill *et al*.^21^
HLA-DRB1*04:04, HLA-DQA1*03:01, HLA-DQB1*03:02	European^c^	34 067 cases & 54 361 controls	0.88,0.89, 0.9	Bellenguez *et al*.^12^
HLA-DRB1*04:04∼HLA-DQA1*03:01∼HLA-DQB1*03:02	European^c^	34 067 cases & 54 361 controls	0.87	Bellenguez *et al*.^12^
HLA-DQA1*03:01, HLA-DQB1*03:02	European^b^	14 776 cases & 23 047 controls	0.9, 0.9	Kunkle *et al*.^22^
HLA-DQB1*06	Iranian	145 cases & 101 controls		Rezaei *et al*.^24^

HLA, human leukocyte antigen; OR, odds ratio.

^a^Odds ratio (OR) values are given, if known.

^b^ADGC—Alzheimer’s Disease Genetics Consortium (U.S.A of European ancestry), EADI—European Alzheimer’s Disease Initiative (France) and GERAD—Genetic and Environmental Risk for Alzheimer’s Disease (Germany, Ireland, U.S.A and U.K.) consortiums.

^c^EADB-TOPMed—combined European Alzheimer’s Disease DNA Biobank (EADB) with Trans-Omics for Precision Medicine (TOPMed) (Belgium, Bulgaria, Czech Republic, Denmark, Finland, France, Germany, Greece, Italy, Portugal, Spain, Netherlands, Sweden, Switzerland, U.K.), GR@ACE/DEGESCO (Spain), GERAD (Germany, U.K., U.S.A.), EADI (France), DemGene (Norway), Bonn (Germany), CCHS—The Copenhagen City Heart Study (Denmark) and EADB-HRC—European Alzheimer’s Disease and Dementia Biobank (EADB) and the Haplotype Reference Consortium (HRC).

Alleles separated by a comma (, ) were tested separately. Alleles separated by a tilde (∼) were tested as a haplotype.

Where OR values are not included in the table, these were either not reported or reported in a different manner.

**Table 2:** Risk HLA haplotypes associated with AD

HLA allele	Population	Cohort size	OR^a^	Reference
HLA-A*02	Southern Chinese	160 cases & 167 controls		Ma *et al*.^25^
HLA-A*02	Chinese	48 cases, 483 MCI & 281 controls		Wang *et al*.^26^
HLA-A*02:01	European^c^	34 067 cases & 54 361 controls	1.03	Bellenguez *et al*.^12^
HLA-DRB1*15:01∼HLA-DQA1*01:02∼HLA-DQB1*06:02∼A*03:01∼B*07:02	European^d^	5728 cases & 5653 controls	1.21	Steele *et al*.^19^
HLA-DRB1*15:01/HLA-DQB1*06:02	Tunisian	55 cases & 100 controls	5.4	Mansouri *et al*.^27^
HLA-DRB1*15:01∼HLA-DQA1*01:01∼HLA-DQB1*06:02	European^b^	14 776 cases & 23 047 controls	1.14	Kunkle *et al*.^22^
HLA-DRB1*03	Caucasian (U.K.)	48 cases and 44 controls	3.8	Neill *et al*.^21^
HLA-DQA1*01:01∼HLA-DQB1*05:01∼HLA-DRB1*01:01	European^c^	34 067 cases & 54 361 controls	1.09	Bellenguez *et al*.^12^
HLA-B*57:01	European^c^	34 067 cases & 54 361 controls	1.11	Bellenguez *et al*.^12^
HLA-B*07	Caucasian (U.K.)	196 cases & 199 controls	2.3	Lehmann *et al*.^28^
HLA-DRB1*09:01	Japanese	303 LOAD & 1717 controls	1.77^e^	Shigemizu *et al*.^29^
HLA-DQB1*03:01	Japanese	303 LOAD & 1717 controls	1.7^e^	Shigemizu *et al*.^29^
HLA-DRB1*04:02∼HLA-DQB1*03:02	Tunisian	55 cases & 100 controls	2.9	Mansouri *et al*.^27^

HLA, human leukocyte antigen; LOAD, Late Onset Alzheimer's disease; MCI, Mild cognitive impairment; OR, odds ratio.

^a^Odds ratio (OR) values are given, if known.

^b^ADGC—Alzheimer’s Disease Genetics Consortium (U.S.A. of European ancestry), EADI—European Alzheimer’s Disease Initiative (France) and GERAD—Genetic and Environmental Risk for Alzheimer’s Disease (Germany, Ireland, U.S.A and U.K.) consortiums.

^c^EADB-TOPMed—combined European Alzheimer’s Disease DNA Biobank (EADB) with Trans-Omics for Precision Medicine (TOPMed) (Belgium, Bulgaria, Czech Republic, Denmark, Finland, France, Germany, Greece, Italy, Portugal, Spain, Netherlands, Sweden, Switzerland, U.K.), GR@ACE/DEGESCO (Spain), GERAD (Germany, U.K., U.S.A.), EADI (France, ), DemGene (Norway), Bonn (Germany), CCHS—The Copenhagen City Heart Study (Denmark) and EADB-HRC—European Alzheimer’s Disease and Dementia Biobank (EADB) and the Haplotype Reference Consortium (HRC).

^d^ADGC—Alzheimer’s Disease Genetics Consortium (U.S.A. of European ancestry).

^e^OR from APOE4 negative individuals.

Alleles separated by a tilde (∼) were tested as a haplotype.

Where OR values are not included in the table, these were either not reported or reported in a different manner.

The largest and most recent AD GWAS identified an intronic SNP rs6605556 near *HLA-DQA1* that associated with reduced risk of AD (*P*-value = 7.1X10^−^,^22^ OR = 0.91). Detailed imputation of the *HLA* locus restricted to clinically diagnosed AD cases reported *HLA-II* risk alleles (DQA1*01:01, DQB1*05:01 and DRB1*01:01), *HLA-II* protective alleles (DQA1*03:01, DQB1*03:02 and DRB1*04:04) and *HLA-I* risk alleles (A*02:01 and B*57:01) in two distinct haplotypes; risk haplotype DRB1*01:01∼ DQA1*01:01∼DQB1*05:01 (OR 1.06–1.14) and protective haplotype DRB1*04:04∼DQA1*03:01∼DQB1*03:02 (OR 0.87).^12,18^ As outlined in Table 1 and Table 2, this recent GWAS of enormous proportions provides evidence in agreement with other previous reports.

Important caveats should be considered when studying *HLA* associations. Firstly, numerous studies cited in Tables 1 and 2 were performed on relatively small samples of subjects, which can often lead to exaggerated effect sizes and false-positives—it is therefore difficult to be confident in many of the associations reported by such smaller studies. Another consideration when comparing *HLA* associations from different studies is the composition of the population they were performed on. For example, the Bellenguez *et al*. GWAS was performed primarily on individuals of European ancestry, which may explain why the differences in *HLA* alleles found differ from those reported in different ethnic populations. Minor discrepancies may also arise due to different methods used for *HLA* typing (e.g. PCR amplification versus *HLA* imputation). Given reports that risk of AD differs in different ethnic populations,^30^ it is clear that future studies will have to expand their genetic inclusions if we are to more fully understand which *HLA* alleles and haplotypes associate with disease risk/protection. Further complexity originates from the fact that *HLA* genes are inherited together as *HLA* haplotypes due to the strong linkage disequilibrium in the region. This means that it can often be difficult to identify causative genes in the region leading to false-positives. Looking for commonalities between *HLA* alleles implicated across genetic backgrounds may contribute to our mechanistic understanding of this association.

### An unconventional role of HLA Class I in the brain?

Investigations into the association of these crucial immune genes with AD have taken place both in humans and in animals. Herein, the term *HLA* will be used to describe evidence in the human context, whilst the term major histocompatibility complex (*MHC*) will be used to describe evidence across species, given each species have their own set of *MHC* genes.

Various hypotheses have been proposed to explain the protective and risk association of *MHC* genes with Alzheimer’s disease. The strongest plausible mechanistic explanation in the context of MHC class I (MHC-I) proteins relates to neuronal homeostatic plasticity with MHC-I expression on neurons shown to be involved in axon regeneration and synaptic pruning.^31^ Mice lacking MHC-I display increased number of synaptic connections between the retina and the receiving lateral geniculate nucleus, with selective re-expression of MHC-I in neurons required to re-establish proper synaptic pruning.^32^ Overexpression of MHC-I in hippocampal neurons resulted in increased elongation and polarization.^33^ This role in the regulation of synaptic pruning has been reported to be dependent on the intracellular domain of MHC-I, implicating intracellular signalling downstream of MHC-I in this process.^34^ This would not be the first observation of classically ‘immune’ proteins having a neurological function; we have previously shown that complement proteins have similarly been implicated in driving pathological synapse loss in AD.^35^ It has also been observed that deletion of the MHC receptor PirB leads to a similar reduction in synaptic pruning,^36^ although it is not clear if MHC-I and PirB interact to mediate this. Given the crucial role of MHC-I and that of a MHC-I receptor in this process, it is not impossible that possession of certain *HLA-I* alleles may make individuals more susceptible to dysregulated synaptic pruning. One potential parallel is the observation that possession of the *HLA-A* allele has been associated with reduced hippocampal volume in AD patients,^26^ although this may stem from other as of yet unknown mechanisms. Highlighting the key role MHC-I plays in the brain, β2-microglobulin (B2M) and TAP double-knockout mice, which lack stable expression of MHC-I on the surface of their cells, display deficits in hippocampal-dependent memory.^37^

MHC-I is known to be expressed on all nucleated cells, with reports confirming expression on neurons,^31^ astrocytes,^38^ oligodendrocytes^39^ and microglia,^40^ although expression seems context dependent. For example, acute viral infection with hepatitis virus in mice has been reported to induce MHC-I expression on oligodendrocytes, neurons and microglial cells.^39^ As such, it is possible that *MHC-I* associations with AD are the result of altered function on cell types other than neurons. One such hypothesis is that HLA-I expression on human brain microvascular endothelial cells is necessary for transendothelial migration of T cells into the brain based on evidence from *in-vitro* blood brain barrier models.^41^ Another possibility is that certain *HLA-I* alleles modulate susceptibility to certain microbial infection, as discussed in the next section.

It is also worth mentioning that MHC-I molecules also display the capacity to bind to a family of receptors expressed on NK cells and T cells known as killer immunoglobulin-like receptors (KIRs). Many KIRs exist, some of which induce activation such as KIR2DS1 and some of which induce inhibition such as KIR2DL1, both of which bind to a subset of HLA-C proteins.^42^ Whilst not the focus of this review, given the breadth of the topic, it is worth highlighting that very little is known about the possible contribution of KIRs on NK cells and T cells and their interaction to HLA-I molecules expressed within the AD brain. To the authors’ knowledge, only one study has explored this, finding that KIR2DS2/KIR2DL2/C1 haplotype was associated with a more severe AD status and increased susceptibility to HHV-6 infection *in-vitro.*^43^ Given this is an area of research being explored in other CNS disease such as multiple sclerosis^44^ and given the experimentation of NK cell based therapies in AD,^45^ this is undoubtedly an interaction worth exploring, perhaps initially at the genetic level to understand whether there are any *KIR* haplotypes associated with AD and how these might interact with *HLA* haplotypes.

In summary, MHC-I proteins have been implicated within the CNS in a function that seems distinct from their role within the immune system, and whilst other mechanisms like regulation of T cell transendothelial migration may be at play, it is clear that synaptic pruning is a ‘low hanging fruit’ that may lead to a better understanding of *HLA-I* associations with AD.

### Does HLA play a role in the infection hypothesis of AD?

Like *HLA-I*, explanation of the observed associations of *HLA-II* haplotypes with AD has not proved straightforward. MHC-II in the brain is known to be expressed by microglial cells, and its expression is increased by inflammatory conditions, such as in the experimental autoimmune encephalomyelitis (EAE) model of multiple sclerosis (MS)^46^ or following exposure to IFN-γ.^47,48^ Beyond microglia, reports also suggest MHC-II expression on astrocytes under similar inflammatory contexts.^38,49,50^ In fact, the majority of AD risk loci are expressed within microglia, including variants that implicate the endolysosomal pathways in AD risk.^12^ This suggests that variants altering all steps of the antigen presentation process, i.e. uptake, processing and presentation on the surface could be sources of AD risk.

At least two hypotheses have been proposed to explain the mechanistic association of *HLA-II* alleles with AD, which may equally hold true for the association with *HLA-I* alleles. The first relates to the infection hypothesis of AD, for which there has been evidence for over 30 years.^51^ This hypothesis proposes that a pathogenic infection is the trigger for the development of AD, with various lines of evidence to support this claim. Vaccination against herpes zoster (HZ) is associated with a reduced incidence of dementia^52^ with further reduced risk when combined with vaccination against tetanus, diphtheria and pertussis.^53^ Higher levels of some herpes virus transcripts have also been found in AD brains relative to control brains.^54^ Evidence from the 3xTg-AD mouse model has also shown that infection with the β-herpesvirus murine cytomegalovirus significantly accelerated cognitive decline and increased the occurrence of tauopathy.^55^ The direct relationship between inflammation and AD is clearly well established and it has been suggested that microbial infection could trigger this inflammatory cascade in AD.^56^ Alternatively, others have proposed that amyloid-β and tau may contribute directly to the immune response within the brain. Amyloid-β possesses anti-microbial properties *in-vitro*^57^ and bacterial DNA have been shown to promote tau aggregation^58^ with a recent study suggesting a direct anti-viral function of tau downstream of the cGAS-STING pathway following infection with HSV1.^59^ These propositions are in line with the observed colocalization of HSV1 DNA within amyloid plaques^60^ and the colocalization of the HSV-1 protein ICP27 and hyperphosphorylated tau aggregates in the brain of AD patients.^59^

As discussed above, polymorphisms at the *HLA* locus and the *HLA* allotype of an individual not only confer risk of developing AD but are known to infer risk/protection to certain infections.^61,62^ A recent study using a cohort of MS patients showed that *HLA* types that have been associated with protection from AD (DRB1*04, DQA1*03:01, DQB1*03:02), are associated with increased titres of anti-Epstein Barr virus (EBV) IgG antibodies.^63^ Similarly, certain SNPs in the *HLA* locus have been implicated in HSV-1 infection.^61^ Therefore, one potential explanation for the association between *HLA* type and AD risk could be that certain *HLA* alleles confer risk/protection during infection with microbes proposed to be involved in the pathogenesis of AD. Exploration of this possibility will require large cohort studies of AD patients and controls, with both infection history and *HLA* type data as exemplified by a recent report that required a cohort of 10 million individuals to provide robust evidence of the association between EBV infection and multiple sclerosis.^64^

### Are AD relevant self-antigens presented via HLA?

An alternative possibility is that certain *HLA* allotypes have the ability to present peptides derived from AD relevant self-antigens. The first line of evidence to support this is the suggestion that protective alleles of the DRB1*04 family present acetylated tau-derived epitopes based on the observation that they bind to acetylated tau PHF6 peptides *in-vitro.*^18^ In parallel, a second study observed that possessing the alleles DQB1*02:01 or DQB1*02:02 was associated with reduced tauopathy in the brain,^65^ although it should be noted that no association of AD with alleles belonging to the DQB1*02 family have previously been reported. A third yet indirect observation is that countries with higher prevalence of DRB1*13:02 display lower rates of dementia.^23^ Given that DRB1*13:02 differs from DRB1*13:01 by a single amino acid that is known to influence the peptide binding groove and in turn the repertoire of presented peptides^66,67^ these observations imply that antigen presentation may be the underlying link between certain *HLA* alleles and AD. Whilst this could be entirely related to risk/protection for certain microbial infections, it also opens up the possibility of the presentation of self-derived AD relevant antigens such as tau being the mechanism of effect.

Whilst the measurement of T cell responses to AD relevant antigens has not generated consistent associations with AD (discussed further below) most studies have measured T cell responses to antigens like amyloid-β or tau in the periphery. It is possible to envision an enrichment of T cells specific for such antigens within the brain itself. How such responses could confer risk/protection from AD is not yet clear. Possible hypotheses include induction of immune responses towards these antigens aiding in their clearance. In fact, an immune response towards Aβ1-42 in a humanized mouse possessing HLA-DRB1*15:01 led to increased clearance of amyloid plaques^68^ and complexes of Aβ42 and MHC-II have been observed on macrophages in mice.^69^ In the context of tau, anti-tau T cell responses could potentially trigger the killing of microglia that have taken up extracellular tau, thereby limiting its cell-cell spreading.^70^ A final possibility is the involvement of tau-reactive regulatory T cells, either brain-resident Tregs^71^ or CD4^+^ T cells poised towards a regulatory phenotype by the microenvironment of the brain. These could help dampen down inflammatory processes within the brain in an antigen-specific manner and, by extension, reduce inflammation related neuronal death, a process that has been suggested to take place in other neurodegenerative conditions like MS.^72^ Equally so, immune responses to such antigens could instead exacerbate inflammation in the brain thereby increasing AD risk. Clearly, many possibilities exist, and untangling the numerous variables at play will not be easy. Understanding the role of HLA in antigen presentation in the AD brain could open numerous immunotherapeutic options, such as those being trialled in other diseases (see Implications section).

### Are antigen-specific T cells enriched in AD patients?

**Figure 2:**
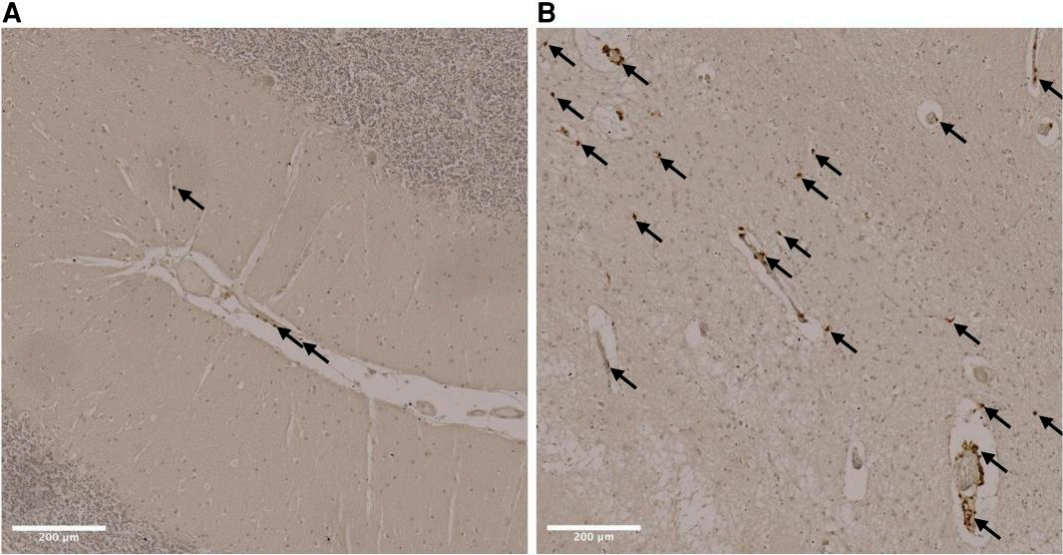
**Evidence of T-cells in the AD brain.** (**A**) Representative immunostaining of formalin fixed paraffin embedded human brain sections, stained following a standard Diaminobenzidine (DAB) staining procedure with a TrisHCl antigen retrieval step using an anti-human CD3E rabbit monoclonal antibody (A19017), specifically (**A**) cerebellum and (**B**) transentorhinal and entorhinal/CA1 from the same individual (BBN006.32514). Arrows highlight the presence of T cells. Scale bar represents 200μm.

Numerous studies have characterized the increased presence of extravascular T cells in the brains of AD patients,^73-76^ specifically in the hippocampus, one of the first regions affected by AD^77^ (Fig. 2). Extravascular T cell numbers have also been reported to correlate with abundance of tau in AD brain but not in control brains.^76^ Brain infiltrating CD8^+^ T cells express higher levels of the cytotoxic proteins granzyme A^78^ and perforin-1^79^ in AD brains relative to healthy control brains, suggestive of a phenotype poised for degranulation. What these T cells may be doing in the brain remains unclear; however, evidence from single cell transcriptomics experiments in aged mice suggests that IFN-γ production by CD8^+^ T cells can inhibit the proliferation of neural stem cells.^80^ Contrasting studies report a protective effect of IFN-γ production by CD4^+^ T cells at the choroid plexus.^81,82^ The infiltration of T cells into the brain has been reported to be facilitated by microglia in AD mouse models; scRNA-seq profiling showed CXCL16-CXCR6 chemokine ligand–receptor interactions between microglia and CD8^+^ T cells in the brain^83^ and depletion of microglia in tauopathy mouse models prevented T cell infiltration and tau pathology.^84^ In humans, close physical interactions between microglia and CD8^+^ T cells have been reported in the human brain^83^ and infiltration of T cells into AD neural-glial cultures induced inflammatory pathways in microglia, including those relating to antigen processing and presentation.^85^ Taken together, the evidence supports a two-way axis between microglia and T cells in both animal models and individuals with AD and suggests that an effector T cell response may be detrimental in AD pathogenesis. These findings have fuelled efforts to further explore effector T cell phenotypes in the context of AD, and it has been observed that AD patients possess increased effector memory CD8^+^ T cells in the blood, a finding that has been replicated by several groups.^78,86,87^ Whether these peripheral changes in T cell phenotype are causative, or the result of disease has yet to be determined.

Another important T cell subset to consider comprises that of regulatory T cells (Tregs). In the 5xFAD mouse AD model, Tregs have been reported to reside in the choroid plexus and, transient depletion of Tregs resulted in recruitment of monocyte-derived macrophages and Tregs themselves into the brain parenchyma with a concomitant reduction in Aβ plaques.^88^ Adoptive transfer of Tregs possessing an Aβ specific transgenic TCR in mice led to reduced microglial activation in the brain.^89^ Treg expansion through administration of low-doses of peripheral IL-2 in AD mouse models, resulted in increased plaque associated microglia^90^ and IL-10 production by cerebral Tregs in rats.^91,92^ These observations suggest that a regulatory T cell response, even directed against AD antigens themselves, could induce protective effects and that Treg function in AD patients may be dysregulated or inadequate. In fact, peripheral Tregs from AD patients are less suppressive *in-vitro,*^92^ an observation that has been replicated.^86^

Studies have also explored peripheral T cell responses to AD relevant antigens, of relevance given the association of AD with *HLA*. Peripheral blood mononuclear cells from older adults and patients with AD showed increased T-cell reactivity to Aβ relative to younger adults.^93^ Furthering this, assessment of T cell responses to a wider pool of AD relevant antigens including tau, Aβ, APP, TDP43 and α-synuclein reported detectable T cell responses against all tested antigens but no significant differences between AD and control groups.^94^ Conversely, a reduced T cell response to Aβ in patients with mild cognitive impairment (MCI) has recently been reported relative to healthy controls.^86^ Importantly, all these studies assessed T cell responses to AD antigens in the periphery, so this does not exclude that such T cell responses could be taking place to a greater degree/with an altered nature locally within the brain/CNS of AD patients. Accordingly, there is: (i) a greater degree of clonal expansion of CD8^+^ T cells in the CSF of AD patients compared to healthy controls,^78^ (ii) an increased clonal expansion of CD4^+^ T cells in CSF of AD patients relative to control individuals^95^ and (iii) tau-mediated clonal T cell expansion in the brain parenchyma in the TE4 mouse model^84^ have all been reported. Combined these studies suggest that AD related T cell responses may be enriched within the brain/CNS. The assessment and comparison of T cell clonality within the human AD brain compared to the healthy brain has not yet been performed, but in combination with improving algorithms that predict TCR epitope specificity from sequence alone,^96^ studies assessing the TCR repertoire within the brain coupled with HLA typing information will likely contribute to our understanding of the nature and role of T cell responses in AD pathology.

### Implications

**Figure 3:**
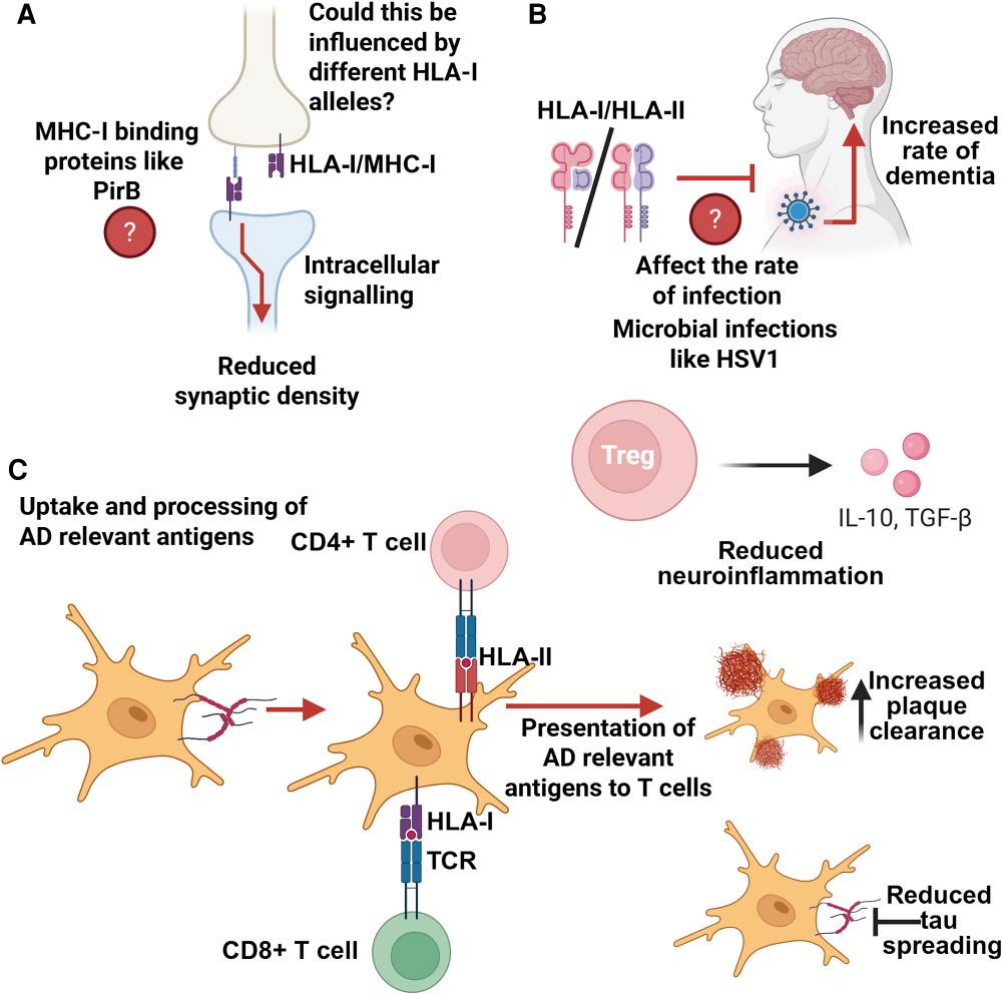
**Summary of hypothesized mechanisms for HLA association with AD.** (**A**) Given the now recognized role played by MHC-I in regulating synaptic plasticity in a manner that involves intracellular signalling from the cytoplasmic domain of MHC-I, it is possible that different alleles may confer a higher risk of dysregulating this mechanism. (**B**) There is now strong evidence to suggest that certain microbial infections may increase the risk of dementia, as such it is possible that certain HLA alleles may increase/decrease the risk of said infections, which may in turn influence the risk of developing dementia. (**C**) Given different HLA alleles present different peptide repertoires, it is possible that alleles may have different propensities for presenting AD relevant antigens, which may in turn alter neuroinflammation in the brain as well as the capacity to clear amyloid plaques and tau neurofibrillary tangles. Created in BioRender. Carpanini, S. (2025) https://BioRender.com/usr8ysg.

Whilst significant work remains, the potential implications of the genetic association of *HLA* alleles with AD are compelling (Fig. 3). At the level of HLA-II, if evidence for specific CD4^+^ T cell responses towards tau (or other antigens) is found in individuals bearing protective *HLA* alleles, this could open the possibility of modulating and expanding such responses. For example, autologous CD4^+^ T cells from AD cases could be captured with HLA specific tau tetramers, expanded and skewed towards a regulatory phenotype *in-vitro* and re-administered to patients, a therapeutic strategy which is being explored in the context of different antigens in type 1 diabetes,^97^ PD^98^ and MS.^99^ Equally, as our understanding of how HLA-I is involved in axonal development and pruning improves, avenues to therapeutically target this pathway could reveal themselves. Finally, a finer understanding of whether individuals carrying certain *HLA* types are more predisposed to microbial infections implicated in AD pathogenesis like HSV1 could aid the development of vaccines with a focus on inducing long-lasting T cells responses.

When considering the strong linkage disequilibrium between *HLA* genes and the parallel extreme degree of polymorphisms observed in this region, it is no surprise that the association of *HLA* with AD has remained a puzzle for so long. Nonetheless, as the availability of high coverage and depth whole exome sequencing and whole genome sequencing approaches become available alongside the plethora of methods to bioinformatically *HLA* type individuals, this association that has until now remained a genetic one may soon translate into an improved understanding of AD disease pathogenesis and form the basis for new therapies.

## Data Availability

Data used in this publication was retrieved from the IMGT-HLA database v3.6.1 and is available at https://www.ebi.ac.uk/ipd/imgt/hla/release/v361/. No additional data was generated for this research. R code employed to collate this data from the IPD-IMGT/HLA database is made publicly available on Github at: https://github.com/LCapitani/HLA_in_AD_review_code.
